# Unknowing ingestion of *Brugmansia suaveolens* leaves presenting with signs of anticholinergic toxicity: a case report

**DOI:** 10.1186/s13256-019-2250-1

**Published:** 2019-10-30

**Authors:** K. P. Jayawickreme, K. V. C. Janaka, S. A. S. P. Subasinghe

**Affiliations:** Sri Jayawardenepura General Hospital, Sri Jayawardenepura Kotte, Sri Lanka

**Keywords:** Solanacea, *Brugmansia suaveolens*, Datura, Atropine, Anticholinergic syndrome

## Abstract

**Background:**

*Brugmansia suaveolens* is the commonest species under the *Solanacea* (“Angels Trumpet” in English; “Attana” in Sinhalese) plant family in Sri Lanka. It contains alkaloids like scopolamine, atropine and hyoscyamine which can cause an anticholinergic toxindrome. There have been a few reported cases of accidental ingestion of *Brugmansia* seeds among children, seeds being the most toxic part, but no such reported cases of *Brugmansia* leaves poisoning among adults.

**Case presentation:**

A 60-year-old-female Sinhalese presented with acute confusion, delirium, and agitation. She had ingested a herbal drink made from leaves of an unknown plant from her garden prior to onset of symptoms. She had urinary retention, mydriasis and sinus tachycardia. She was managed supportively with activated charcoal and hydration and the delirium completely resolved within 15 hours. The presented unkown plant leaves were identified as *Brugmansia suaveolens.*

**Conclusion:**

Although seeds are the most toxic plant part in most cases of *Brugmansia* poisoning, leaves also have a significant degree of toxicity. It is important that medical professionals promptly recognize the features of anticholinergic syndrome, and have a high index to suspect *Brugmansia* poisoning and start prompt treatment. It is also important to improve awareness of toxic plants among the general community to prevent toxicities and fatalities.

## Introduction

Self-poisoning carries a high mortality and morbidity in developing countries like Sri Lanka. The German regional poison control center showed 9.7% of poisoning cases to be due to plant poisoning, indicating its global burden [1]. It carries a high burden on hospital admissions, with a case fatality ratio of 9% in Sri Lanka but is highly under-evaluated [2]. Ingestion of pesticides used to be the commonest mode of poisoning in Sri Lanka, but now is less frequent and has a change in trend towards drug overdose causing poisoning as a result of regulations and restricting availability to pesticides. However this has a geographical variation, with 49% hospital admissions to North Central province rural hospitals being due to pesticides, and 34% due to orleander poisoning [2]. A study in the National hospital of Sri Lanka which assessed the pattern in the western province showed 68% of poisoning cases to be due to medicinal drugs, 21% due to pesticides, and 6.28% due to orleander poisoning [3]. 2.5% of poisoning cases were due to plant poisoning, and yellow orleander poisoning being the commonest accounting for 17% cases in Sri Lanka [4, 5]. Cases of *Brugmansea* poisoning in adults were not reported in Sri Lanka so far to our knowledge. Cases of *Datura* or *Brugmansia* poisoning in other countries were almost always due to ingestion of seeds [6]. Since the rarity of presentation due to this rare toxic plant leaf, the knowledge of it’s toxicity may be poor among the population, and doctors may not have a high index of suspicion in identifying such cases.

## Case presentation

A 60-year-old Sinhalese housewife with pre-existing hypertension and diabetes mellitus presented to the emergency treatment unit with acute confusion, delirium and agitation. She had prepared and drank 350 ml of a herbal drink containing leaves in her garden, which the other family members were not aware of. She routinely prepared herbal drinks from other plants in her garden, and was unaware of this plant’s toxicity. She was previously well until she developed acute confusion, agitation and restlessness 30 minutes after the drink. Another family member who also had the same drink in a lesser quantity of 50 ml had developed mild confusion which recovered spontaneously within 6 hours. She had not taken any other medication other than her routine drugs; losartan 25 mg and metformin 850 mg. She had no history of psychiatric illness and had no similar episodes in the past, and denies taking any other drugs other than her routine medications. She did not smoke or consume alcohol.

On examination she was disoriented in time, place, and person and was restless. She had mydriasis and dry skin. Her blood pressure was 140/90 mmHg, compared to her baseline blood pressure of 120/80 mmHg, and had a tachycardia of 120 beats per minute. She had acute urinary retention, which drained 1000 ml of dilute urine after catheterization. She was afebrile, had no neck stiffness, no papilledema, and no focal neurological signs, and limb tone, power and reflexes were normal. She had no cerebellar signs and no sensory impairment. Her random blood sugar was 150 mg/dl, electrocardiogram (ECG) showed sinus tachycardia, and venous blood gas showed a PH of 7.42, lactate- 1.5 mmol/l, HCO^−^ _3_ 24 mmol/l. Her computed tomography (CT) Brain was normal. Her serum creatinine was 70 mmol/L, serum sodium was 135 mmol/L, serum potassium was 4.3 mmol/L. Urinalysis had no pus cells or red cells. Her Alanine Aminotransferase was 20 U/L, Aspartate aminotransferase was 32 U/L, and International normalized ratio was 1. Her C reactive protein was < 2 mg/L. Her urine for toxicology was negative for illicit drug substances like amphetamine and cocaine.

**Fig. 1:**
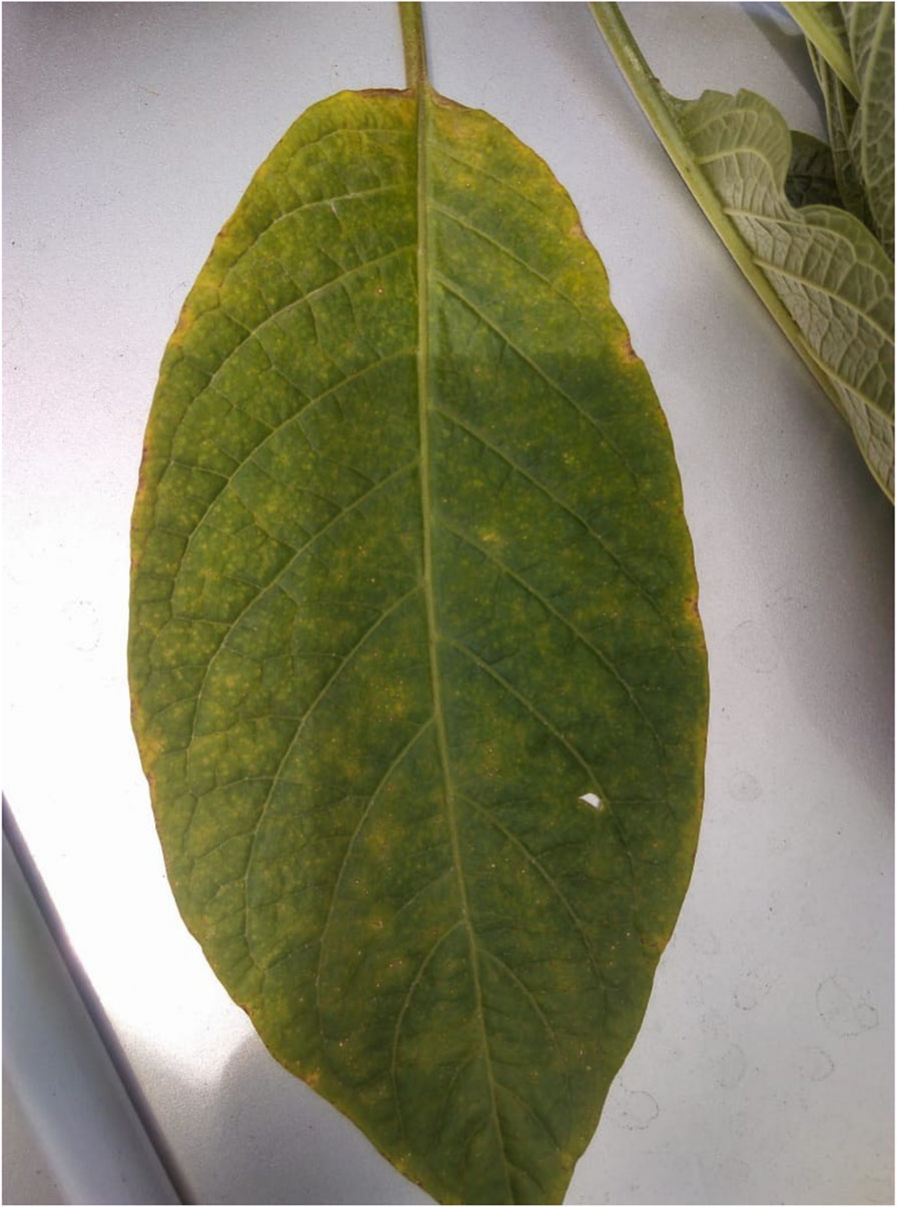
Leaf of *Brugmansia suaveolens* plant from the patients garden which was mixed in the kanji drink

**Fig. 2:**
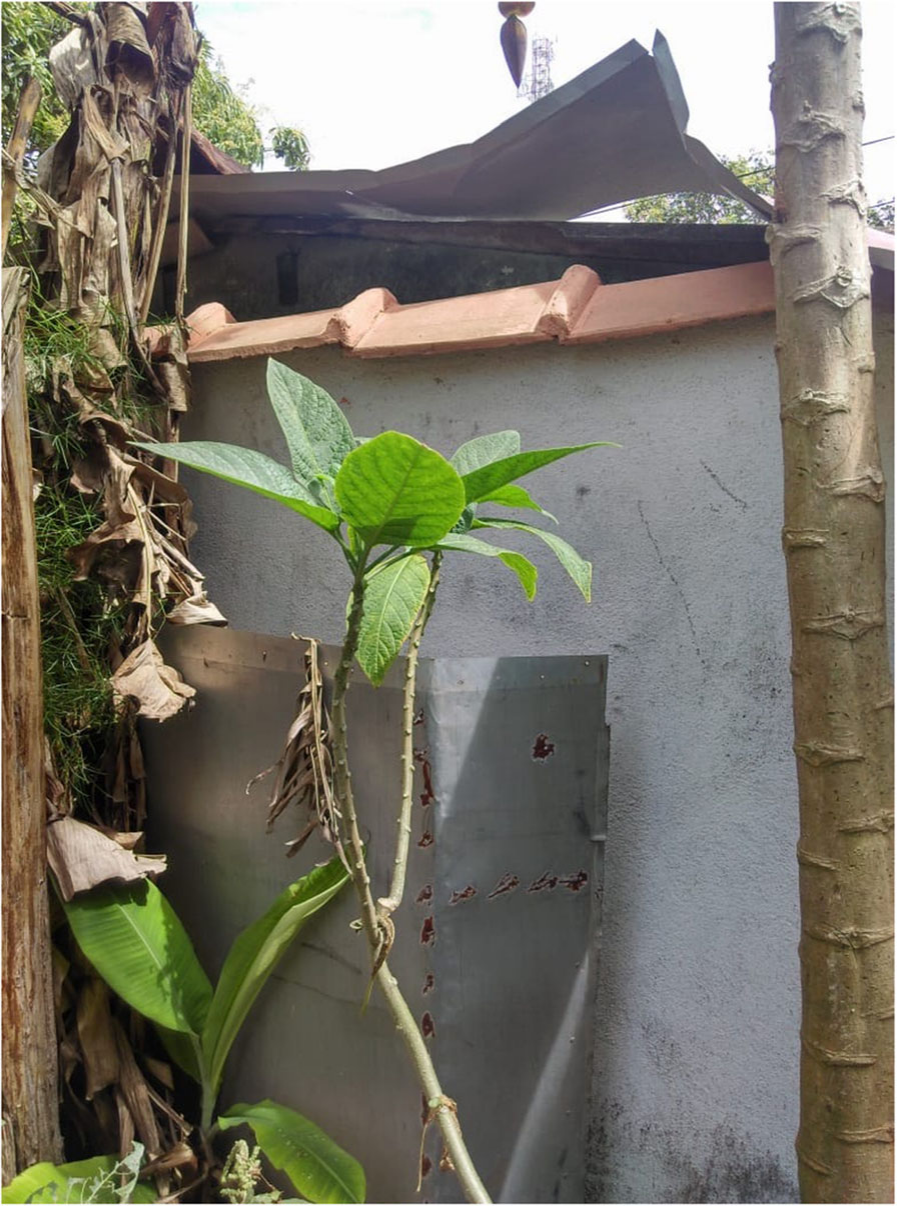
The young *Brugmansia suaveolens* plant from the patients garden. Its flowers have not bloomed yet

Since she presented 6 hours after the presumed poisoning of an unknown toxin, gastric lavage was not done, but multiple dose activated charcoal was given, assuming the possibility of features of anticholinergic syndrome causing delayed gastric emptying. She was well hydrated with normal saline and 1.5 mg IV midazolam was given to calm the patient. Her delirium gradually weaned off after 15 hours since onset and mydriasis settled after 24 hours, and recovered without any residual effect. After regaining consciousness she admitted that she made the kanji sample containing *Centella asiatica* leaves (“Gotukola” in sinhalese), *Asparagus racemosus* (“Hathawariya” in sinhalese), and another unknown plants leaves from her garden. We got down a part of the unknown plant (Fig. 1) and images of the tree (Fig. 2), and with the help of a native medicine physician and specialist in botany, identified the plant as a species of “Attana”; *Brugmansia suaveolens*. However she did not require the antidote physostigmine and recovered fully. On discharge she and her family members were educated on the toxicity of the plant and were advised to avoid ingestion of any parts of the plant. She was completely normal at follow up at 6 months.

## Discussion

We present a rare case of unknowing ingestion of *Brugmansia suaveolens* leaves in an adult presenting with signs of anticholinergic toxicity. This is a rare presentation due to ingestion of leaves of this palnt, and thus needs a high index of suspicion in such cases.

*Datura* and *Brugmansia* are two genera under the family *Solanacea,* collectively referred to as “Attana” in Sinhala language. *Brugmansia suaveolens* is the commonest species under this family in Sri Lanka. They contain alkaloids like scopolamine, atropine and hyoscyamine which have an anticholinergic effect. It has been used in the past to sedate sacrificial victims in spiritual ceremonies and as an anaesthetic agent for external use [7]. There have been reported cases of intentional ingestion of *Brugmansia* species by adolescents for recreational purposes due to its hallucinogenic and euphoric effect. Toxicity causes confusion, hallucinations, tachycardia, hyperthermia, dryness, urinary retention, mydriasis and death [7, 8].

The two main genera in the family *Solanacea* are differentiated in their appearance by plants of the *Datura* genus being mainly Bushes with their flowers facing upwards; giving them the name “Devils Trumpet”, and plants of the *Brugmansia* genus mainly being trees with their flowers facing downwards; giving them the name “Angels Trumpet” [9]. These plants are grown for ornamental use and many are unaware of its toxic nature as in this case.

The degree of toxicity varies depending on the part of the plant, the season, stage of maturation and the state of hydration. Toxicity can occur via ingestion, smoking, and absorption topically, particularly through mucous membranes. All parts of the plant can be toxic, but most significantly the seeds. The seeds of *Datura stramonium*; which is a species under the *Solanacea* family, contains 0.1 mg of atropine per seed, or 3–6 mg per 50–100 seeds [8]. Each blossom of *Brugmansia* contains 0.65 mg scopolamine and 0.3 mg atropine. There are reported fatalities at atropine doses of 10 mg, which accounts for ingestion of as few as 10 flowers [8]. The flowers may contain 0.83% hyoscine, and 0.4% hyoscine in the leaves. The flowers of older plants are more toxic containing 3 mg of hyoscine [10]. The age of this plant was about one year, indicating moderate toxicity. However the toxic constituents in both *Datura* and *Brugmansia* are mostly the same, being alkaloids causing an anticholinergic effect by blocking the muscarinic acetylcholine receptors of the central and peripheral nervous system.

A hospital based prospective study among Children in Sri Lanka with plant poisoning in 2006 reported 7 cases of *Datura stramonium* poisoning [11]. There were no such published reported cases of *Brugmansia suaveolens* poisoning among adults in Sri Lanka. This patient presented with features of anticholinergic toxindrome; hyperpyrexia, dryness of skin and mucous membranes, mydriasis, confusion, agitation, hallucinations, tachycardia and urinary retention. A study in Australia which assessed the clinical effects of *Brugmansia* poisoning in adults showed mydriasis to occur in 100% cases, delirium in 88% cases and tachycardia in 33% cases [12]. A study in Sri Lanka among children with *Datura* poisoning showed hyperpyrexia, mydriasis, and tachycardia to occur in 100% cases, delirium in 71% cases and hallucinations in 14% cases [11]. Respiratory failure and cardiovascular collapse have been reported in severe cases [13]. Severe agitation can cause Rhabdomyolysis leading to acute kidney injury, also predisposed by direct toxicity of the alkaloids, which we prevented by adequate hydration of the patient. *Datura stramonium* toxicity usually occurs within 60 minutes since ingestion, with symptoms lasting upto 24–48 hours due to delayed gastric emptying and fat solubility. Children have a higher susceptibility to atropine toxicity causing worse effects with smaller amounts of toxin [14]. A study on effects of *Brugmansia* poisoning showed mydriasis to last for a mean duration of 29 hours, and delirium to last for 18 hours [12]. This patient developed symptoms 30 minutes after ingestion of the toxin, and delirium gradually improved within about 15 hours and mydriasis improved after about 24 hours of onset. She had mixed *Brugmansia* leaves with “Gotukola” (*Centella asiatica)* and “Hathawariya” (*Asparagus racemosus*) in the drink, which are commonly consumed. Interstingly both *Centella asiatica and Asparagus racemosus* have an anti-cholinesterase effect which antagonizes the effect of *Brugmansia,* but did not neutralize the effect as the toxicity and toxic dose of *Brugmansia* was probably more significant than the antagonist [15, 16].

Management is mostly supportive. This patient presented 6 hours after ingestion of the toxin, so it was too late to do gastric lavage, but activated charcoal was given as gastric emptying is delayed by the anticholinergic effect of the toxin. Acute urinary retention was relieved by catheterization, and was well hydrated. Benzodiazepines were given to control agitation. Phenothiazines for agitated delirium should be avoided due to its anticholinergic properties, and barbiturates can be administered for seizures refractory to benzodiazepines [13]. The antidote for anticholinergic toxicity is physostigmine which is a cholinesterase inhibitor, but its use is controversial despite recent reports of its safe use. However physostigmine is relatively contraindicated in cardiac conduction defects. Physostigmine is only recommended in severe cases of agitation or psychosis refractory to benzodiazepines, intractable seizures or coma, or tachyarrhythmias with hemodynamic compromise. Administration of physostigmine was not required in management of this case [13, 17]. This patient had a good outcome and recovered completely.

We reported a case of high anticholinergic toxicity by accidental ingestion of *Brugmansia suaveolens* leaves, without being aware of its toxic effects. Further research can be recommended based on the knowledge of toxic plants in the Sri Lankan community, and measures must be taken to improve awareness among the general public of toxic effects of plants and recognizing such plants by their appearance.

## Conclusion

Although seeds are the most toxic plant part in most cases of *Brugmansia* and *Datura* poisoning, it’s leaves also have significant toxicity. It is important that medical professionals promptly recognize the features of anticholinergic syndrome in patients who present following unknown plant poisoning, and have a high index to suspect *Brugmansia* or *Datura* poisoning and start prompt treatment to improve outcome. It is also important to improve awareness of toxic plants among the general community to prevent toxicities and fatalities.

## Data Availability

The data and raw material is available in case if needed.
